# Willingness to use and preferences for long‐acting injectable PrEP among sexual and gender minority populations in the southern United States, 2021–2022: cross‐sectional study

**DOI:** 10.1002/jia2.26077

**Published:** 2023-03-23

**Authors:** Paige Schoenberg, O. Winslow Edwards, Leland Merrill, Cristian Acero Martinez, Rob Stephenson, Patrick S. Sullivan, Jeb Jones

**Affiliations:** ^1^ Department of Epidemiology Emory University Atlanta Georgia USA; ^2^ Department of Systems Populations and Leadership School of Nursing and The Center for Sexuality and Health Disparities, University of Michigan Ann Arbor Michigan USA

**Keywords:** sexual and gender minority populations, pre‐exposure prophylaxis, HIV, injectable, men who have sex with men, transgender people

## Abstract

**Introduction:**

Long‐acting injectable (LAI) pre‐exposure prophylaxis (PrEP) for HIV prevention was approved by the U.S. Food and Drug Administration in 2021. LAI PrEP is more effective than oral PrEP. However, it is not clear whether the groups most at risk of HIV in the United States will use LAI PrEP. Willingness to use LAI PrEP and preference for LAI versus oral PrEP has not been reported for sexual and gender minority (SGM) people in the southern United States, where the HIV epidemic is concentrated. Our goal was to assess willingness to use LAI PrEP and preference for oral versus LAI PrEP among SGM people in the southern United States and to assess differences in willingness by demographics and sexual behaviour.

**Methods:**

We conducted an online, cross‐sectional survey of SGM people aged 15–34 years in the southern United States (*n* = 583). Participants reported willingness to use LAI PrEP and preferences for LAI PrEP versus daily oral PrEP. We assessed bivariate associations and adjusted prevalence ratios for the LAI‐PrEP‐related outcomes and key demographic and behavioural characteristics.

**Results:**

Overall, 68% of all participants (*n* = 393) reported being willing to use LAI PrEP that provides protection against HIV for 3 months. Of those, most (*n* = 320, 81%) indicated a preference for using LAI PrEP, compared to a daily oral pill or no preference. Willingness to use LAI PrEP was more common among transgender and non‐binary participants and participants who engaged in condomless anal intercourse in the last 6 months. Hispanic participants were more likely and non‐Hispanic Black participants were less likely to report willingness to use LAI PrEP compared to non‐Hispanic White participants.

**Conclusions:**

Willingness to use LAI PrEP was high among SGM people in the southern United States, although there were some important differences in willingness based on demographic characteristics. Decreased willingness to use LAI PrEP among groups who are disproportionately affected by the HIV epidemic, such as non‐Hispanic Black SGM people, could exacerbate existing disparities in HIV incidence. LAI PrEP is an acceptable option among SGM populations in the southern United States, but strategies will be needed to ensure equitable implementation.

## 1 INTRODUCTION

In December 2021, the U.S. Food and Drug Administration (FDA) announced the approval of a novel extended‐release form of the HIV prevention drug cabotegravir for use as a long‐acting injectable (LAI) pre‐exposure prophylaxis (PrEP) [1]. Cabotegravir is the first LAI option for HIV prevention, approved for use by cisgender men, cisgender women, and transgender women [1]. Daily oral PrEP adherence can be a challenge [2, 3], a problem that might be mitigated by LAI PrEP because LAI PrEP is administered via intramuscular injections every 2 months [1]. Among cisgender men and transgender women who have sex with men, LAI PrEP reduces HIV risk by 66–69% compared to oral PrEP [4].

Prior to FDA approval of injectable cabotegravir, a handful of studies investigated the acceptability of LAI PrEP. These studies, primarily focused on coastal urban populations, for example [5, 6], found LAI PrEP to be acceptable and even preferable to oral PrEP among cisgender gay, bisexual, and other men who have sex with men (GBMSM) [5, 6]. Fewer data are available for transgender and gender‐expansive populations; however, qualitative data suggest interest in LAI PrEP among these populations [7]. Among GBMSM and gender minority populations, more HIV diagnoses occur in the South than in any other region of the United States [8] and most HIV diagnoses in rural areas are among GBMSM [9]. In the South and outside of urban centres, there is a possibility that the acceptability of LAI PrEP may be lower, possibly because barriers to use, including experiences of stigma in healthcare settings [10], may be greater.

There are multiple reasons that LAI PrEP might be preferred over oral PrEP. It requires less frequent dosing, which might help overcome the challenges that daily dosing poses for many people [11]. People in violent relationships in which other prevention measures might be difficult to use could potentially use LAI PrEP without their partners’ knowledge [12]. However, potential PrEP users might also have concerns about receiving frequent injections and question the long‐term protection provided by LAI PrEP [6, 13].

The objective of this study was to assess the acceptability of LAI PrEP among sexual and gender minority (SGM) people in urban and non‐urban areas in the South. We also aimed to describe differences in willingness to use LAI PrEP and preference for PrEP modality based on demographics, including race and ethnicity, gender identity, age, socioeconomic characteristics, and sexual health history.

## 2 METHODS

### 2.1 Study population

We conducted an online survey of SGM people aged 15–34 years who live in the southern United States. This age group is at the highest risk of HIV infection in the United States. Recruitment and data collection took place between April and January 2022. The study adhered to federal human subjects regulations and was reviewed and approved by Emory University's human subjects institutional review board (protocol IRB001268). The requirement of parental assent was waived for participants less than 18 years old.

Participants were recruited through convenience sampling via online social and sexual networking sites (e.g. Instagram, Grindr and Jack'd). On social networking sites (e.g. Instagram), ads were additionally targeted towards users whose social media activity indicated they were likely to belong to an SGM group. Eligible participants completed informed consent electronically and were able to begin the survey immediately. Survey topics included demographics; sexual behaviours; substance use; HIV and sexually transmitted infection testing, attitudes, and beliefs; and use of HIV prevention services. Participants were not initially incentivized for participation; however, a weekly raffle for a $50 electronic gift card was initiated in October 2021. The survey was hosted on HIPAA‐compliant servers at Alchemer.com.

Participants were eligible if they were assigned male at birth regardless of gender identity or assigned female at birth and identified as transgender or non‐binary, were age 15–34, resided in the southern United States as defined by the United States Census Bureau, had an Android or iOS phone with active service and were willing to download a study app to their phone, spoke English, had ever had anal or vaginal/frontal sex, and reported being HIV negative at their last HIV test or never having been tested for HIV. Cisgender men were required to report a history of anal sex to be eligible in order to exclude cisgender men who have sex with women only.

### 2.2 Measures

Participants were first shown the following text: “A new form of PrEP that is delivered via an injection and provides protection against HIV for three months is currently being studied. This is sometimes called long‐acting injectable (LAI) PrEP.” The 3‐month dosing frequency was based on the initial inter‐injection interval for LAI PrEP and differs from the 2‐month interval that was approved in December 2021. Participants were first asked if they would be willing to use LAI PrEP. Response options included yes, no, and not sure, which were dichotomized as yes or no/not sure for analysis. Participants were also asked, “Would you prefer to take LAI PrEP that would provide protection against HIV for three months, or daily oral PrEP?” Response options included LAI PrEP, daily oral PrEP, and no preference, which were dichotomized for analysis as preferring LAI PrEP or preferring oral PrEP/having no preference. To isolate the preference based on the method of administration, we did not provide data regarding the efficacy of the different PrEP modalities.

Independent measures included rurality of residence, gender identity, age, education level, annual household income before taxes, health insurance status, condomless anal intercourse (CAI) within the last 6 months, current PrEP use, and feelings of stigma towards PrEP use. Rurality was based on the Index of Relative Rurality (IRR) rural classification system based on county of residence [14]. IRR values ≥ 0.4 were considered to be rural based on recommendations from the developers of the scale [15] and a prior study demonstrating that this cutoff effectively differentiates rural and non‐rural GBMSM [16]. Feelings of stigma towards PrEP use was a derived variable created by summing dichotomized answers to Likert scale questions about stigmatized attitudes towards PrEP. Participants were asked to agree or disagree with five statements about PrEP and stigma, adapted from questions previously used to assess sexually transmitted disease‐related stigma [17]. Participants who answered, “strongly agree” or “agree” were categorized as “agree” and participants who answered, “neutral,” “disagree” or “strongly disagree” were categorized as “disagree.” This dichotomized the responses into groups who endorsed and who did not endorse each stigmatizing statement. We decided a priori to classify those who agreed with two or more out of five statements as having feelings of stigma towards PrEP use.

### 2.3 Statistical analyses

We assessed bivariate associations between the demographic characteristics of the study population and the outcomes of willingness to use LAI PrEP and preference for the PrEP method using Fisher's exact test. We also assessed the overlap between willingness to use LAI PrEP and preference for LAI PrEP.

Binomial logistic regression using predicted margins standardization was used to estimate unadjusted and adjusted prevalence ratios (aPRs) [18] for the outcomes of willingness to use LAI PrEP and preferred form of PrEP. Adjusted models included rurality of residence, gender identity, age, education level, annual household income before taxes, health insurance status, engaging in CAI within the last 6 months, history of oral PrEP use (current, past, never) and feelings of stigma towards PrEP use. Statistical analyses were conducted using SAS v9.4.

## 3 RESULTS

There were 583 eligible participants recruited from 16 southern states and Washington, DC. The states contributing the most participants were Texas (*n* = 127), Georgia (*n* = 79) and Virginia (*n* = 57); the states contributing the fewest were Washington, DC (*n* = 11), West Virginia (*n* = 6) and Delaware (*n* = 1). After removing participants with missing or invalid data on rurality, willingness to use PrEP, and annual household income, the final analytic sample included 575 study participants. Most participants (68%) were from non‐rural areas (Table 1). Most identified as cisgender male, and 16% identified as transgender or non‐binary. Participants were most commonly college graduates or higher and had health insurance. Nearly all participants reported engaging in CAI in the last 6 months. Thirty‐six percent of participants reported ever using PrEP and 23% of participants were currently using PrEP.

**Table 1:** Willingness to use long‐acting injectable PrEP and select demographic characteristics of sexual and gender minority survey respondents in the southern United States, 2021

	Total	Yes	Not sure or not willing	Unadjusted prevalence ratio (PR) and 95% confidence interval (CI)	Adjusted prevalence ratio and 95% confidence interval
	*N*	*n* (%)	*n* (%)	PR (95% CI)	PR (95% CI)
	575	393 (68%)	182 (32%)		
**Rurality**					
Non‐rural	392	272 (69%)	120 (31%)	Ref	Ref
Rural	183	121 (66%)	62 (34%)	0.95 (0.84, 1.08)	1.02 (0.90, 1.16)
**Race/ethnicity**					
Hispanic	105	79 (75%)	26 (25%)	1.10 (0.96, 1.26)	1.07 (0.92, 1.24)
Non‐Hispanic Black	129	75 (58%)	54 (42%)	0.85 (0.72, 1.00)	0.86 (0.73, 1.03)
Non‐Hispanic White	292	200 (69%)	92 (32%)	Ref	Ref
Other/multiracial	47	39 (83%)	8 (17%)	1.21 (1.04, 1.41)	1.22 (1.05, 1.42)
**Gender identity**					
Cisgender male	481	325 (68%)	156 (32%)	Ref	Ref
Transgender, non‐binary or other	93	67 (72%)	26 (28%)	1.07 (0.93, 1.23)	1.21 (1.05, 1.39)
**Age group**					
15–24 years	175	125 (71%)	50 (29%)	Ref	Ref
25–34 years	400	268 (67%)	132 (33%)	0.94 (0.83, 1.05)	0.92 (0.81, 1.04)
**Education level**					
High school or lower	109	69 (63%)	40 (37%)	Ref	Ref
At least some college	465	323 (69%)	142 (31%)	1.10 (0.94, 1.28)	1.17 (0.96, 1.42)
**Annual household income before taxes**					
$0–$19,999	135	86 (64%)	49 (36%)	Ref	Ref
$20,000–$39,000	145	100 (69%)	45 (31%)	1.08 (0.91, 1.28)	1.04 (0.89, 1.23)
$40,000–$74,999	140	98 (70%)	42 (30%)	1.10 (0.93, 1.30)	1.05 (0.88, 1.24)
$75,000 or more	115	79 (69%)	36 (31%)	1.08 (0.90, 1.29)	0.98 (0.80, 1.19)
**Insurance status**					
None	131	96 (73%)	35 (27%)	Ref	Ref
Public or private insurance	439	292 (67%)	147 (33%)	0.91 (0.80, 1.03)	0.88 (0.78, 1.00)
**Condomless anal intercourse in the last 6 months**					
No	89	49 (55%)	40 (45%)	Ref	Ref
Yes	432	318 (74%)	114 (26%)	1.34 (1.10, 1.63)	1.23 (1.03, 1.48)
**Stigmatizing views of PrEP use**					
No	537	371 (69%)	166 (31%)	Ref	Ref
Yes	38	22 (58%)	16 (42%)	0.84 (0.63, 1.11)	0.89 (0.71, 1.13)
**History of oral PrEP use**					
None	363	232 (64%)	131 (36%)		Ref
Current	130	102 (79%)	28 (22%)	1.23 (1.09, 1.38)	1.16 (1.01, 1.32)
Past	77	56 (73%)	32 (27%)	1.14 (0.97, 1.33)	1.10 (0.93, 1.30)

Sixty‐eight percent of all participants reported willingness to use LAI PrEP that provides protection against HIV for 3 months. Of those currently using PrEP, 79% were willing to use LAI PrEP, compared to 73% of those who had used PrEP in the past and 64% of those who had never used PrEP. Demographic characteristics that were most strongly associated with willingness to use LAI PrEP were race/ethnicity and gender identity. Rurality was not associated with willingness to use LAI PrEP. Non‐Hispanic Black participants were less likely than other groups to be willing to use LAI PrEP. In the adjusted model, non‐Hispanic Black participants were 14% less likely to be willing to use LAI PrEP compared to non‐Hispanic White participants (aPR = 0.86, 95% confidence interval [CI]: 0.73, 1.03). Hispanic participants (aPR = 1.07, 95% CI: 0.92, 1.24) and participants of other or multiple races (aPR = 1.22, 95% CI: 1.05, 1.39) had a higher willingness to use LAI PrEP compared to non‐Hispanic White participants.

Behavioural characteristics were also associated with willingness to use LAI PrEP. Participants who reported CAI in the past 6 months had a higher willingness to use LAI PrEP compared to those who reported no CAI (aPR = 1.23, 95% CI: 1.03, 1.48). Current oral PrEP use (aPR = 1.16, 95% CI: 1.01, 1.32) and past oral PrEP use (aPR = 1.14, 95% CI: 0.93, 1.30) were also associated with greater willingness to use LAI PrEP.

Among the demographic characteristics examined, only race/ethnicity and educational attainment were associated with a preference for LAI PrEP compared to daily oral PrEP or having no preference (Table 2). Hispanic (aPR = 1.26, 95% CI: 1.02, 1.55), non‐Hispanic Black (aPR = 1.17, 95% CI: 0.94, 1.44), and other or multiracial (aPR = 1.52, 95% CI: 1.23, 1.89) participants were all more likely to prefer LAI PrEP compared to non‐Hispanic White participants. CAI and current PrEP use were not associated with preference for LAI PrEP.

**Table 2:** Preference for long‐acting injectable PrEP versus daily oral PrEP or no preference among sexual and gender minority survey respondents in the southern United States, 2021

	Total	Prefer LAI PrEP	Daily pill or no preference	Unadjusted prevalence ratio (PR) and 95% confidence interval (CI)	Adjusted prevalence ratio and 95% confidence interval
	*N*	*n* (%)	*n* (%)		
	575	320 (56%)	255 (44%)		
**Rurality**					
Non‐rural	392	221 (56%)	171 (44%)	Ref	Ref
Rural	183	99 (54%)	84 (46%)	0.96 (0.82, 1.13)	1.10 (0.94, 1.30)
**Race/ethnicity**					
Hispanic	105	52 (59%)	43 (41%)	1.13 (0.93, 1.38)	1.26 (1.02, 1.55)
Non‐Hispanic Black	129	70 (54%)	59 (46%)	1.04 (0.86, 1.26)	1.17 (0.94, 1.44)
Non‐Hispanic White	292	152 (52%)	140 (48%)	Ref	Ref
Other/multiracial	47	35 (74%)	12 (26%)	1.43 (1.17, 1.75)	1.52 (1.23, 1.89)
**Gender identity**					
Cisgender male	481	267 (56%)	214 (44%)	Ref	Ref
Transgender, non‐binary or other	93	53 (57%)	40 (43%)	1.03 (0.85, 1.25)	1.11 (0.88, 1.41)
**Age group**					
15–24 years	175	96 (55%)	79 (45%)	Ref	Ref
25–34 years	400	224 (56%)	176 (44%)	1.02 (0.87, 1.20)	1.00 (0.83, 1.21)
**Education level**					
High school or lower	109	50 (46%)	59 (54%)	Ref	Ref
At least some college	197	270 (58%)	195 (42%)	1.27 (1.02, 1.58)	1.31 (0.98, 1.74)
**Annual household income before taxes**					
$0–$19,999	135	71 (53%)	64 (47%)	Ref	Ref
$20,000–$39,000	145	78 (54%)	67 (46%)	1.02 (0.82, 1.28)	0.97 (0.77, 1.23)
$40,000–$74,999	140	77 (55%)	63 (45%)	1.05 (0.84, 1.30)	1.02 (0.80, 1.31)
$75,000 or more	115	74 (64%)	41 (36%)	1.22 (0.99, 1.51)	1.12 (0.87, 1.44)
**Insurance status**					
None	131	71 (54%)	60 (46%)	Ref	Ref
Public or private insurance	439	247 (56%)	192 (44%)	1.04 (0.87, 1.24)	1.01 (0.84, 1.23)
**Condomless anal intercourse in the last 6 months**					
No	89	45 (51%)	44 (49%)	Ref	Ref
Yes	432	249 (58%)	183 (42%)	1.14 (0.91, 1.42)	1.03 (0.83, 1.27)
**Stigmatizing views of PrEP use**					
No	537	299 (56%)	238 (44%)	Ref	Ref
Yes	38	21 (55%)	17 (45%)	0.99 (0.74, 1.34)	1.00 (0.74, 1.34)
**History of oral PrEP use**					
None	363	186 (51%)	177 (49%)	Ref	Ref
Current	130	79 (61%)	51 (39%)	1.19 (1.00, 1.41)	1.10 (0.90, 1.34)
Past	77	51 (66%)	26 (34%)	1.29 (1.07, 1.56)	1.30 (1.07, 1.58)

Among those who were willing to use LAI PrEP, 278 (*n* = 71%) indicated a preference for LAI PrEP (Table 3).

**Table 3:** Preference for long‐acting injectable PrEP by willingness to use long‐acting injectable PrEP among sexual and gender minority survey respondents in the southern United States, 2021

		Preferred PrEP modality	
	Total	LAI PrEP	Daily pill or no preference
	*N*	*n* (%)	*n* (%)
	575	320	255
**Willing to use LAI PrEP**			
Yes	393	278 (71%)	115 (29%)
No	182	42 (23%)	140 (77%)

## 4 DISCUSSION

We observed a high willingness to use LAI PrEP and a preference for LAI PrEP over daily oral PrEP among SGM people in the southern United States. There were differences in LAI PrEP willingness based on race/ethnicity, gender identity, and behavioural characteristics including history of PrEP use and history of CAI.

In addition to being highly effective, LAI PrEP mitigates issues of non‐adherence frequently associated with daily oral PrEP [2, 3]. FDA approval of LAI PrEP is an encouraging development in HIV prevention, but only if those with PrEP indications are willing to use it. It is thus a positive sign for PrEP expansion efforts that two‐thirds of participants in our study said they would use LAI PrEP. Our findings are in line with previous studies, such as one 2017 study of GBMSM in Washington, DC, which found that 62% of GBMSM were interested in LAI PrEP [5].

We found important differences in willingness to use LAI PrEP by race/ethnicity, gender identity, and socio‐economic characteristics. Seventy‐five percent of Hispanic participants were willing to use LAI PrEP, compared to just 58% of non‐Hispanic Black participants. These numbers are similar to findings from a study in Washington, DC, which found that 68% of Hispanic participants and 61% of non‐Hispanic Black participants were interested in LAI PrEP [5]. Non‐Hispanic Black GBMSM are consistently less willing to use or less interested in LAI PrEP, likely due in part to a long history of medical mistrust among Black Americans stemming from a history of systemic racism in medicine and public health [19, 20, 21, 22]. Reduced willingness to use LAI PrEP among Black SGM people has the potential to further exacerbate existing disparities in HIV incidence.

Transgender or non‐binary participants were 20% more likely than cisgender male participants to be willing to use LAI PrEP. Transgender men and women are at high risk for HIV [23], and this increased willingness might reflect existing engagement with the healthcare system that would facilitate receiving injections.

Our study provides important evidence of the feasibility of studying PrEP preferences among rural SGM people. We were able to recruit a racially diverse sample of participants with substantial rural participants, most of whom identified as something other than non‐Hispanic White. The HIV epidemic in the United States is concentrated among non‐Hispanic Black and Hispanic people, predominantly GBMSM [24]. Understanding the willingness to use LAI PrEP among these groups is one key step to understanding potential barriers to implementation. We were also able to recruit a relatively large number of transgender or non‐binary participants, who comprised 16% of the study population.

Our study is subject to common limitations. The sample was a convenience sample of participants primarily recruited via social and sexual networking websites and apps. Thus, our study population is not representative of all SGM populations in the southern United States. Additionally, the study asked participants about PrEP preferences without considering possible deterrents to LAI PrEP, such as cost. Cost differences will likely play an important role in the uptake of LAI versus oral PrEP; the effect of cost on uptake should be an ongoing focus of studies as LAI PrEP is implemented. We asked participants to consider an LAI PrEP that required injections every 3 months, whereas the FDA‐approved injectable cabotegravir is given every 2 months. Instead of requiring four injections annually, the drug requires six injections, which may deter potential users. However, in a sample of urban GBMSM, LAI PrEP was the most preferred option when described as requiring an injection every 1–3 months [25], indicating that the difference between every 2 months and every 3 months might not substantially affect preferences for LAI PrEP.

## 5 CONCLUSIONS

Our study, conducted just before FDA approval of injectable cabotegravir, found LAI PrEP to be an acceptable option among GBMSM in the southern United States. Most SGM people were both willing to use LAI PrEP and preferred it over daily oral pills, or they had no preference for the PrEP form. LAI PrEP may be of particular interest to those who wish to protect themselves against HIV but have trouble adhering to a daily pill. However, additional interventions or information campaigns focused towards non‐Hispanic Black men and those with a high school education or less may be necessary to increase the uptake of LAI PrEP among these groups.

## COMPETING INTERESTS

The authors have no competing interests to disclose.

## AUTHORS’ CONTRIBUTIONS

JJ, RS and PSS conceived of the study. JJ, OWE, LM, PSS and RS designed the study. PS, CAM and JJ contributed to data analyses. PS and JJ wrote the manuscript. All authors read and approved the final manuscript.

## FUNDING

This study was funded by the National Institute of Nursing Research (R56NR019482).

## Data Availability

Data are available by request to the corresponding author.
